# Age-related nomogram revealed optimal therapeutic option for older patients with primary liver cancer: less is more

**DOI:** 10.18632/aging.205901

**Published:** 2024-06-06

**Authors:** Bo Wang, Yongqiang Xiong, Ren Li, Shu Zhang

**Affiliations:** 1 Department of Geriatric Digestive Surgery, The Second Affiliated Hospital of Xi’an Jiaotong University, Xi’an, China; 2 Experimental Teaching Center for Clinical Skills, The Second Affiliated Hospital of Xi’an Jiaotong University, Xi’an, China

**Keywords:** primary liver cancer, age, dynamic nomogram, SEER, therapeutic options

## Abstract

Background: Age bias in therapeutic decisions for older patients with cancer exists. There is a clear need to individualize such decisions.

Methods: Based on the Surveillance, Epidemiology and End Results (SEER) database, 5081 primary liver cancer (PLC) patients between 2010 and 2014 were identified and divided into <64, 64-74 and >74 years group. Each group was randomly divided into training and internal validation cohorts, and patients who were diagnosed between 2015 and 2016 were included as an external validation. The nomogram model predicting overall survival (OS) was generated and evaluated based on the Cox regression for the influencing factors in prognosis. The K-M analysis was used to compare the difference among different treatments.

Results: KM analysis showed a significant difference for OS in three age groups (*P* < 0.001). At the same time, we also found different prognostic factors and their importance in different age groups. Therefore, we created three nomograms based on the results of Cox regression results for each age group. The c-index was 0.802, 0.766, 0.781 respectively. The calibration curve and ROC curve show that our model has a good predictive efficacy and the reliability was also confirmed in the internal and external validation set. An available online page was established to simplify and visualize our model (http://124.222.247.135/). The results of treatment analysis revealed that the optimal therapeutic option for PLCs was surgery alone.

Conclusions: The optimal therapeutic option for older PLCs was surgery alone. The generated dynamic nomogram in this study may be a useful tool for personalized clinical decisions.

## INTRODUCTION

Primary liver cancer (PLC) is a common cancer of the digestive tract, and the most dominant risk factors include liver cirrhosis, viral hepatitis, consumption of aflatoxin-contaminated foods, etc. [1]. Histologically, PLC mainly includes hepatocellular carcinoma (HCC) (75% -85%) and intrahepatic cholangiocarcinoma (ICC) (10% -15%), as well as other rare types. According to the Global Cancer Statistics 2020, primary liver cancer was the sixth most commonly diagnosed malignant cancer globally with 906,000 new cases comprising 4.7% of total new cases in 2020. Due to its poor prognosis, it was the third leading cause of cancer death with 830,000 deaths, 8.3% of the total cancer deaths [2]. Currently, the main treatments for PLCs were surgery, including resection and liver transplantation, chemotherapy, local radiotherapy, and combination therapy. Moreover, systemic therapeutic approaches have been approved, including immunotherapy such as atazolizumab combined with bevacizumab, and targeted therapies including checkpoint inhibitors as well as tyrosine kinase inhibitors [3]. Despite this, the 5-year survival rate of PLCs was only 30% - 50% and the prognosis still remained poor [4]. Consequently, the choice of an appropriate therapeutic option holds paramount importance for primary liver cancer.

Throughout the world, with an increasing life expectancy, many countries are facing challenges associated with an aging population. It is obvious that the average age at diagnosis of PLC has been increasing in many developed countries. A report suggested that the average age at diagnosis of HCC increased from 67.1 years (1998-2002) to 69.1 years (2013-2016). Furthermore, the proportion of patients who aged 70 and above at diagnosis rose from 39.6% (1998-2002) to 47.5% (2013-2016) [5]. For most chronic diseases, including PLC, age stands as a significant factor contributing to a poor prognosis. A recent Japanese report demonstrated that older patients (≥75 years old) had significantly worse overall survival (OS) after surgical excision compared to younger patients [6, 7].

Nomogram is a simple and accurate visualization tool based on the multivariate analysis. It is utilized to predict and quantify the survival of each patient and to guide the clinical decisions [8, 9]. In this study, we developed and validated three nomograms for prognostic prediction and risk stratification of PLCs in different age groups. Furthermore, we evaluated the therapeutic strategy in each age group based on data obtained from the National Cancer Institute’s Surveillance, Epidemiology, and End Results (SEER) program.

## MATERIALS AND METHODS

### Data source and patient selection

The data were obtained from National Cancer Institute’s Surveillance, Epidemiology, and End Results (SEER) program between 2010 and 2016. SEER serves as an open-access resource providing demographic, clinicopathological, and some treatment information related to tumors. All the data used in this study were retrieved from the SEER*Stat Version 8.4.0 (http://www.seer.cancer.gov/seerstat). As the study utilized publicly available data, local ethical approval and declaration were not required.

The inclusion criteria were as follows: 1) the patients were diagnosed between 2010 and 2016; 2) primary tumor were located in the liver and intrahepatic bile duct; 3) there was only one primary tumor and the behavior was malignant. The exclusion criteria were: 1) incomplete patient demographics, including race and marital status; 2) incomplete clinicopathological characteristics, such as grade, AJCC TNM stage, tumor size, AFP level, metastasis status, survival information, and treatment details.

### Clinical variables of patients

The information of demographics included age at diagnosis, sex, marital status (categorized as married and unmarried, including separated, divorced, widowed, single or domestic partner), race (white, black, and others, including American Indian/Alaska Native, Asian/Pacific Islander). The tumor-related factors included tumor size (<=5, 5-10, >10 cm), grade, AFP level, histology, AJCC TNM stage, and metastatic status involving bone, brain, and lung. The therapeutic options were categorized as follows based on the codes from the SEER program: no treatment (N), surgery alone (S), chemotherapy alone (C), radiation alone (R), surgery combined with chemotherapy (S+C), surgery combined with radiation (S+R), chemotherapy combined with radiation (C+R), and surgery combined with chemotherapy and radiation (S+C+R). The primary endpoint of interest was overall survival (OS), defined as the time from the date of the first-time diagnosis until the date of death caused by any cause or the most recent follow-up.

### Construction of nomogram

We divided all patients into three groups according to age at diagnosis using the X-tile software. The X-tile software is a new bio-informatics tool for biomarker assessment and outcome-based cut-point optimization, obtained from Yale University School of Medicine, USA. The X-tile offers a single, global assessment of each possible way of dividing the population into low, middle and high level of marker expression [10]. For the construction of nomogram, the patients who were diagnosed between 2010 and 2014 from the SEER program were randomly divided into a training set and an internal validation set at a ratio of 7:3. The patients who were diagnosed between 2015 and 2016 from the SEER program were used as an external validation set. The training set was used to construct the nomogram, and its efficacy was assessed through internal and external validation sets.

In the training cohort, the independent risk factors were identified by univariate and multivariate Cox proportional-hazards regression analyses for OS [11]. A backward stepwise method, based on the smallest Akaike information criterion (AIC) value, was finally employed to incorporate the covariates into the multivariate Cox proportional hazards models. The AIC value suggests the minimal loss of prognostic information [12, 13]. Cox regression and the nomogram were developed by using the ‘rms’ package in R software. The predictive discrimination ability of the nomogram was assessed by using C-index and the area under curve (AUC) [14, 15]. Calibration curves-based boots method was utilized to examine the association between actual OS and predicted OS by the nomogram in the training and validation set [16]. The decision curve analysis (DCA) was also developed to estimate the clinical utility and benefits of the prediction model [17, 18]. We divided patients into three risk groups according to the total score of each patient and the Kaplan-Meier survival curve with a log-rank test was used to verify the value of the risk classification [19]. Additionally, an interactive dynamic nomogram web page was built to visualize our results using ‘DynNom’ package [20].

### Statistical analysis

Statistical analysis was performed by using R software version 4.1.2. The difference among groups was compared using the Pearson’s chi-square test or Fisher’s exact test for categorical variables appropriately. Survival curve was compared using the log-rank test. *P* < 0.05 was considered statistically significant.

### Data availability

The data of this study are available in the SEER database (https://seer.cancer.gov/).

## RESULTS

### Study cohort characteristics

**Figure 1:**
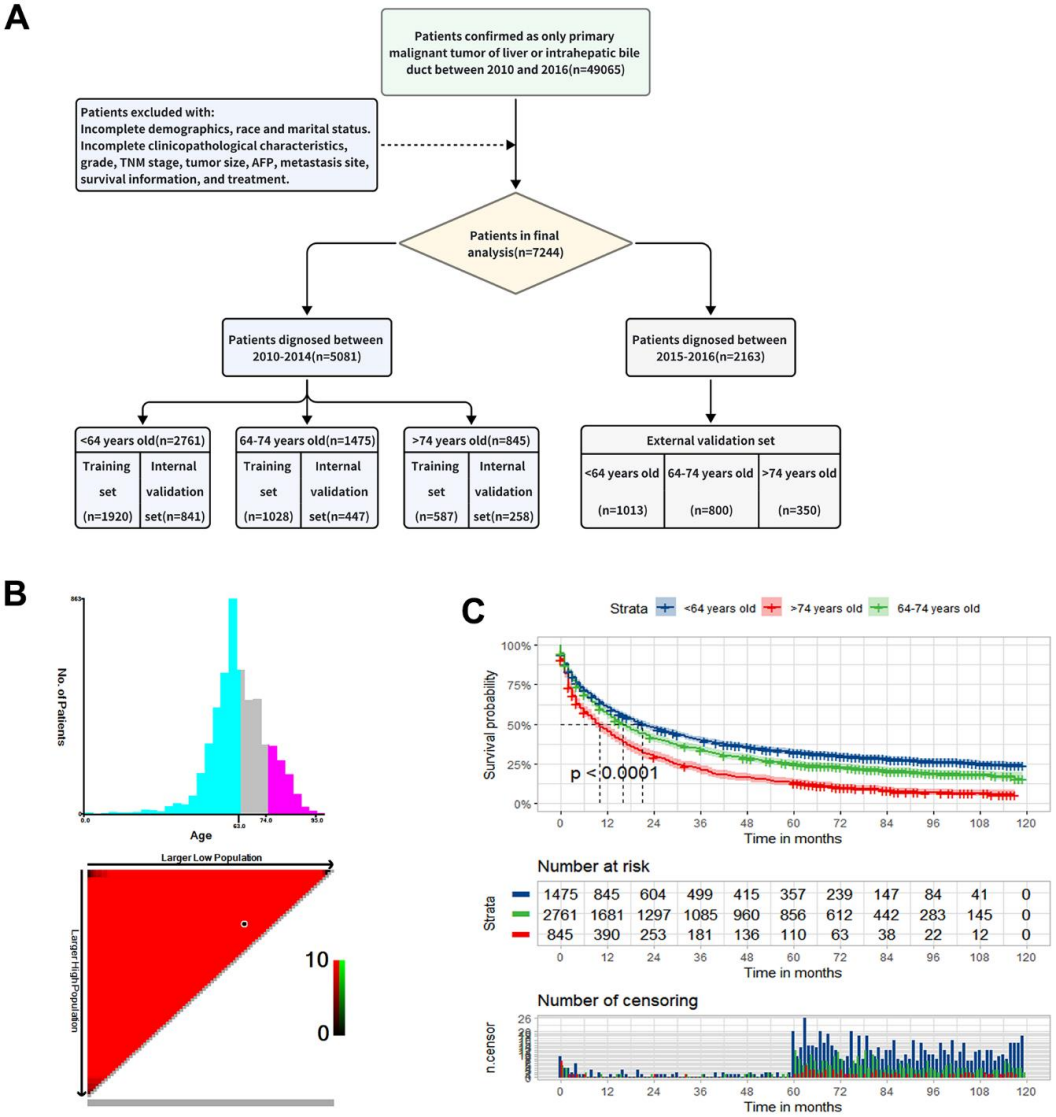
**Study design and patient selection.** (**A**) The study flow diagram. (**B**) The optimal age cut-off point for overall survival (OS) defined with X-tile software. (**C**) The Kaplan-Meier curve for overall survival among the low, middle and old age groups.

We ultimately included a total of 7,244 eligible PLCs from the SEER database based on the above inclusion and exclusion criteria. The flowchart of the patient selection process is shown in Figure 1A. Generally, the majority of patients were male (73.9%), white (67.6%), married (57.8%), AFP positive (64%), T1 (42.7%), N0 (89.5%), M0 (86.5%) stage and the common histology is HCC (91%). 46% of tumors was moderately differentiated/Grade II. The lung represented the most common site of metastasis, accounting for 5% of cases (Table 1).

**Table 1:** The baseline data of patients with primary liver cancer diagnosed at 2010-2014.

**Characteristics**		**<64 years old (n=2761, %)**	**64-74 years old (n=1475, %)**	**>74 years old (n=845, %)**	**P-value**
Sex	Male	2174 (78.7)	1056 (71.6)	523 (61.9)	<0.001
	Female	587 (21.3)	419 (28.4)	322 (38.1)	
Race	White	1844 (66.8)	1002 (67.9)	588 (69.6)	<0.001
	Black	400 (14.5)	165 (11.2)	50 (5.9)	
	Other	517 (18.7)	308 (20.9)	207 (24.5)	
Marital	Married	1537 (55.7)	889 (60.3)	509 (60.2)	0.004
	Unmarried	1224 (44.3)	586 (39.7)	336 (39.8)	
Histology	HCC	2550 (92.4)	1336 (90.6)	767 (90.8)	0.090
	ICC	211 (7.6)	139 (9.4)	78 (9.2)	
Tumor.size	<5cm	1454 (52.7)	646 (43.8)	248 (29.3)	<0.001
	5—10cm	813 (29.4)	546 (37.0)	372 (44.0)	
	>10cm	494 (17.9)	283 (19.2)	225 (26.6)	
Grade	Grade I	672 (24.3)	374 (25.4)	240 (28.4)	0.166
	Grade II	1291 (46.8)	676 (45.8)	374 (44.3)	
	Grade III	757 (27.4)	396 (26.8)	212 (25.1)	
	Grade IV	41 (1.5)	29 (2.0)	19 (2.2)	
T.stage	T1	1130 (40.9)	647 (43.9)	392 (46.4)	<0.001
	T2	753 (27.3)	321 (21.8)	145 (17.2)	
	T3	745 (27.0)	419 (28.4)	262 (31.0)	
	T4	133 (4.8)	88 (6.0)	46 (5.4)	
N.stage	N0	2445 (88.6)	1326 (89.9)	775 (91.7)	0.026
	N1	316 (11.4)	149 (10.1)	70 (8.3)	
M.stage	M0	2380 (86.2)	1275 (86.4)	740 (87.6)	0.591
	M1	381 (13.8)	200 (13.6)	105 (12.4)	
Treatment	N	581 (21.0)	362 (24.5)	318 (37.6)	<0.001
	S	871 (31.5)	433 (29.4)	183 (21.7)	
	C	716 (25.9)	417 (28.3)	245 (29.0)	
	R	56 (2.0)	50 (3.4)	44 (5.2)	
	S+C	384 (13.9)	120 (8.1)	22 (2.6)	
	S+R	12 (0.4)	12 (0.8)	3 (0.4)	
	C+R	112 (4.1)	69 (4.7)	27 (3.2)	
	S+C+R	29 (1.1)	12 (0.8)	3 (0.4)	
AFP	Negative	945 (34.2)	535 (36.3)	339 (40.1)	0.007
	Positive	1816 (65.8)	940 (63.7)	506 (59.9)	
Bone metastasis	No	2681 (97.1)	1426 (96.7)	829 (98.1)	0.136
	Yes	80 (2.9)	49 (3.3)	16 (1.9)	
Brain metastasis	No	2750 (99.6)	1474 (99.9)	843 (99.8)	0.125
	Yes	11 (0.4)	1 (0.1)	2 (0.2)	
Lung metastasis	No	2627 (95.1)	1405 (95.3)	798 (94.4)	0.651
	Yes	134 (4.9)	70 (4.7)	47 (5.6)	
Months (median [IQR])		20.0 [5.0,67.0]	16.0 [4.0,56.5]	9.0 [2.0,29.0]	<0.001

We further stratified the patients who diagnosed between 2010 and 2014 (n=5081) into three groups namely low-age group (n=2761): <64 years old, middle-age group (n=1475): 64-74 years old, old-age group (n=845): >74 years old, according to the best cut-off value based on the X-tile (Figure 1B). The Kaplan-Meier survival curve for OS showed significant difference in different age groups (*P* < 0.001) (Figure 1C). There was no doubt that the younger patients have better OS and the median OS of three groups was 20,16 and 9 months, respectively (Figure 1C and Table 1). Also, there was significant difference among three groups in terms of sex, marital status, race, tumor size, T stage, N stage, AFP level and therapeutic options in Table 1. In the low-age group, the most common tumor size was less than 5 cm, while it was 5-10 cm in old-age group. In the low and middle-age group, surgery alone (S) was the most treatment option, followed by chemotherapy alone (C), but in the old-age group, 38% of patients did not receive any treatment (N). Another cohort comprising 2,163 patients diagnosed between 2015 and 2016 was selected to as an external validation set. The demographics and clinicopathological characteristics were summarized in Supplementary Table 1.

### Low-age group

The total 2761 patients in this group were divided randomly into the training set (n=1920) and the internal validation set (n=841). The baseline characteristics of the group is shown in Supplementary Table 2. The 1-, 3-, and 5-year OS rates were 60.9%, 39.3%, and 31.0% in the initial set. There was no significant difference between distribution of these variables in the training and internal validation sets. The 1,013 patients who diagnosed between 2015 and 2016 were selected as an external validation set.

**Figure 2:**
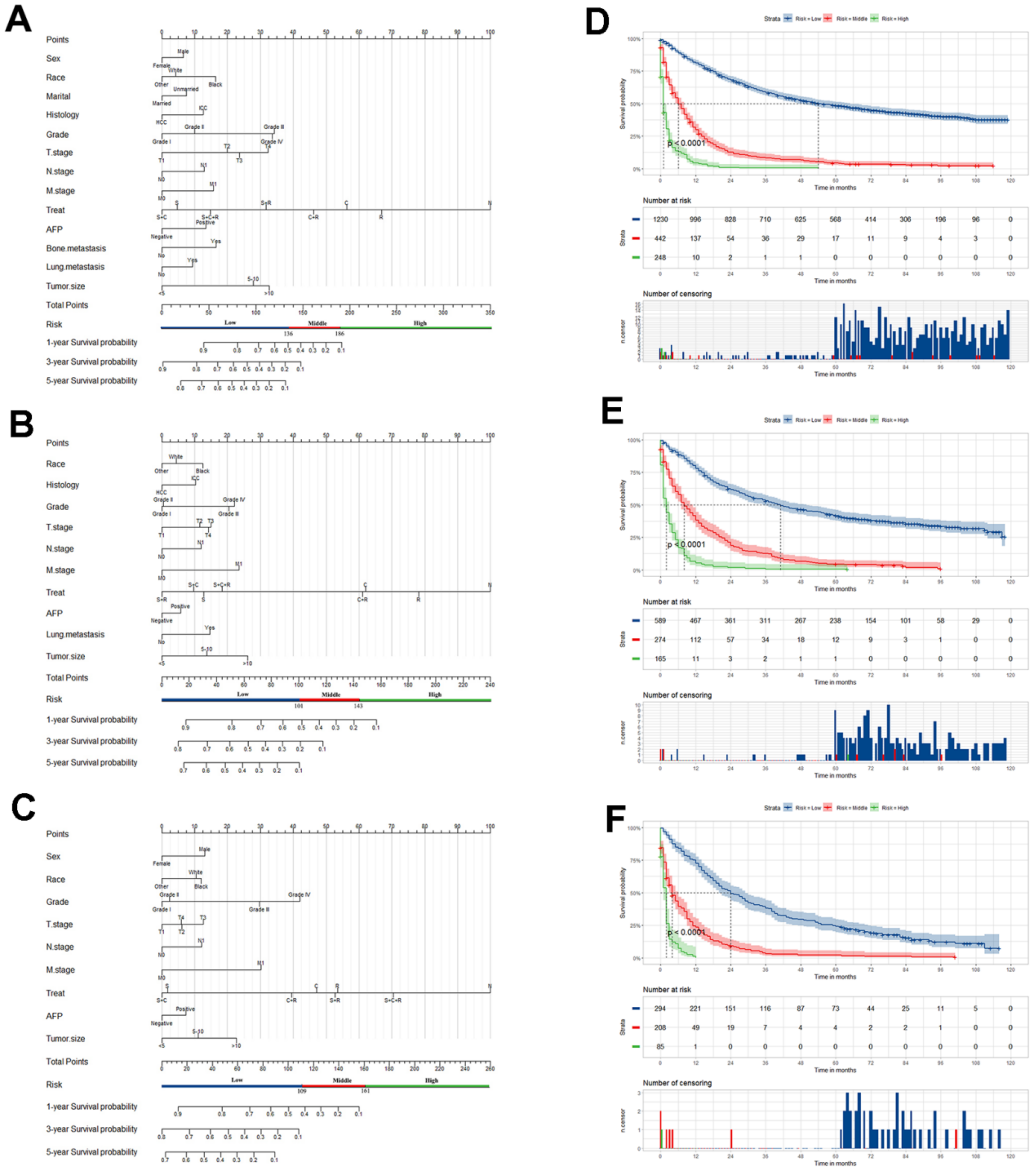
**Nomogram of prognosis prediction in primary liver cancer patients (PLCs).** Nomogram for predicting the overall survival (OS) of PLC patients in the low- (**A**), middle- (**B**) and old- (**C**) age group, respectively. Kaplan-Meier curves for OS among the low-risk, middle-risk, and high-risk groups stratified according to the Nomo-score in the low- (**D**), middle- (**E**) and old- (**F**) age group, respectively.

As illustrated in Table 2, the univariate Cox analysis conducted in the training set showed that the significant indicators were sex, race, marital status, histology, grade, tumor size, T stage, N stage, M stage, AFP level, treatment and bone, brain, lung metastasis. The model yielded the smallest AIC value (AIC=17734.15) when including the aforementioned 13 independent indicators into the multivariate Cox analysis, except brain metastasis. Based on the model, we constructed a satisfactory nomogram for the prediction of 1-, 3- and 5-year OS probability of PLCs in low-age group (Figure 2A). Each variable has a specific value on the points scale, and the total Nomo-score was calculated by summing these scores to predict the 1-, 3- and 5-year survival probability for individual patient.

**Table 2:** Univariate and multivariate Cox regression of the training set in low-age group.

**Characteristics**		**Univariate Cox analysis**			**Multivariate Cox analysis**		
		**HR**	**95%CI**	**p-value**	**HR**	**95%CI**	**p-value**
Sex	Male	R			R		
	Female	0.85	0.74-0.97	0.018	0.87	0.76-1	0.053
Race	White	R			R		
	Black	1.42	1.22-1.64	<0.001	1.26	1.08-1.47	0.003
	Other	0.82	0.71-0.95	0.007	0.92	0.79-1.07	0.271
Marital	Married	R			R		
	Unmarried	1.37	1.23-1.52	<0.001	1.16	1.04-1.3	0.008
Histology	HCC	R			R		
	ICC	1.52	1.27-1.82	<0.001	1.29	1.05-1.59	0.016
Tumor.size	<5cm	R			R		
	5—10cm	2.5	2.21-2.82	<0.001	1.75	1.5-2.04	<0.001
	>10cm	3.11	2.7-3.58	<0.001	1.94	1.63-2.31	<0.001
Grade	Grade I	R			R		
	Grade II	1.05	0.91-1.21	0.499	1.21	1.05-1.41	0.009
	Grade III	2.3	1.99-2.67	<0.001	2.03	1.73-2.39	<0.001
	Grade IV	2.42	1.6-3.68	<0.001	1.96	1.27-3.01	0.002
T.stage	T1	R			R		
	T2	1.18	1.02-1.36	0.022	1.53	1.32-1.78	<0.001
	T3	3.35	2.94-3.82	<0.001	1.66	1.43-1.94	<0.001
	T4	3.9	3.1-4.9	<0.001	2.03	1.58-2.6	<0.001
N.stage	N0	R			R		
	N1	2.89	2.48-3.36	<0.001	1.32	1.12-1.56	0.001
M.stage	M0	R			R		
	M1	3.73	3.24-4.3	<0.001	1.4	1.14-1.72	0.001
Treatment	N	R			R		
	S	0.11	0.1-0.13	<0.001	0.26	0.15-0.45	<0.001
	C	0.47	0.41-0.54	<0.001	0.55	0.24-1.29	0.171
	R	0.47	0.34-0.66	<0.001	1.09	0.43-2.77	0.862
	S+C	0.13	0.1-0.16	<0.001	0.32	0.1-1.08	0.066
	S+R	0.24	0.12-0.48	<0.001	NA	NA	NA
	C+R	0.48	0.37-0.63	<0.001	NA	NA	NA
	S+C+R	0.26	0.16-0.42	<0.001	NA	NA	NA
AFP	Negative	R			R		
	Positive	1.5	1.34-1.69	<0.001	1.35	1.19-1.54	<0.001
Bone metastasis	No	R			R		
	Yes	3.71	2.77-4.98	<0.001	1.31	0.93-1.84	0.126
Brain metastasis	No	R			R		
	Yes	5.49	2.28-13.22	<0.001	1.98	0.78-5	0.15
Lung metastasis	No	R			R		
	Yes	3.6	2.89-4.49	<0.001	1.21	0.93-1.58	0.164

**Figure 3:**
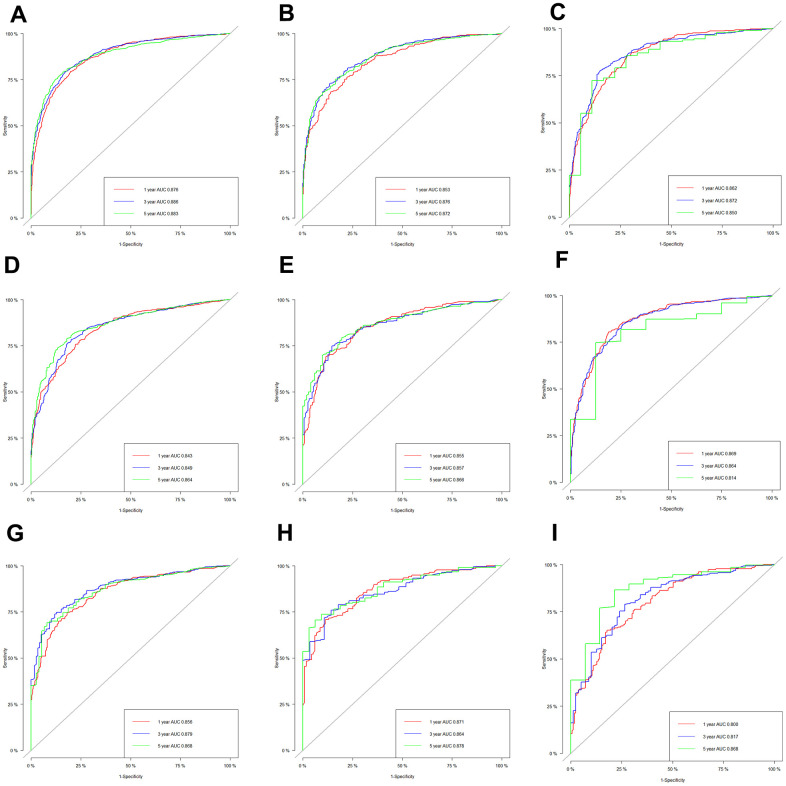
**The receiver operating characteristic (ROC) curves based on nomogram.** The ROC curves based on nomogram in the training, internal and external validation set of low-age group (**A**–**C**), middle-age group (**D**–**F**) and old-age group (**G**–**I**).

The AUC values of the nomogram to predict 1-, 3- and 5-year OS were 0.876 (95% CI, 0.860-0.891), 0.886 (95% CI, 0.871-0.901) and 0.883 (95% CI, 0.868–0.899) in the training set, respectively (Figure 3A). In the internal validation set, the AUC values to predict 1-, 3- and 5-year OS were 0.853 (95% CI, 0.826-0.879), 0.876 (95% CI, 0853-0.899) and 0.872 (95% CI, 0847-0.896), respectively (Figure 3B). In the external validation set, the AUC values to predict 1-, 3- and 5-year OS were 0.862 (95% CI, 0.839-0.885), 0.872 (95% CI, 0.849-0.895) and 0.850 (95% CI, 0.760-0.939), respectively (Figure 3C). The C-index of nomogram were 0.802, 0.786 and 0.797 in the training, internal validation and external validation sets.

### Middle-age group

In this group, 1475 patients were randomly divided into the training set (n=1028) and internal validation set (n=447). The information was summarized in Supplementary Table 3. Similarly, there was no significant difference between distribution of these variables in the training and internal validation sets. The 800 patients who diagnosed between 2015 and 2016 were selected as an external validation set. In Table 3, the univariate Cox analysis in the training set showed the significant indicators. The model yielded the smallest AIC value (AIC=9526.1) in the multivariate Cox analysis with 10 variables: race, primary site, grade, tumor size, T stage, N stage, M stage, AFP level, treatment and lung metastasis. We constructed a nomogram to predict the 1-, 3- and 5-year OS probability of PLCs in middle-age group (Figure 2B).

**Table 3:** Univariate and multivariate Cox regression of the training set in middle-age group.

**Characteristics**		**Univariate Cox analysis**			**Multivariate Cox analysis**		
		**HR**	**95%CI**	**p-value**	**HR**	**95%CI**	**p-value**
Sex	Male	R			R		
	Female	0.80	0.68-0.93	0.005	0.92	0.78-1.09	0.360
Race	White	R			R		
	Black	1.20	0.98-1.48	0.082	1.17	0.95-1.46	0.147
	Other	0.76	0.63-0.91	0.003	0.93	0.77-1.12	0.426
Marital	Married	R			R		
	Unmarried	1.29	1.12-1.48	<0.001	1.09	0.94-1.27	0.266
Histology	HCC	R			R		
	ICC	1.56	1.25-1.95	<0.001	1.27	0.98-1.63	0.068
Tumor.size	<5cm	R			R		
	5—10cm	1.89	1.61-2.21	<0.001	1.33	1.1-1.62	0.004
	>10cm	2.34	1.93-2.84	<0.001	1.76	1.4-2.21	<0.001
Grade	Grade I	R			R		
	Grade II	0.84	0.71-1	0.046	1.03	0.86-1.23	0.761
	Grade III	1.42	1.18-1.71	<0.001	1.57	1.29-1.91	<0.001
	Grade IV	1.48	0.94-2.34	0.093	1.63	1.02-2.61	0.042
T.stage	T1	R			R		
	T2	1.02	0.84-1.23	0.838	1.29	1.05-1.58	0.015
	T3	2.34	1.98-2.76	<0.001	1.4	1.16-1.7	0.001
	T4	2.45	1.85-3.23	<0.001	1.38	1.02-1.86	0.035
N.stage	N0	R			R		
	N1	2.47	2-3.07	<0.001	1.31	1.02-1.67	0.032
M.stage	M0	R			R		
	M1	3.53	2.91-4.29	<0.001	1.61	1.22-2.13	0.001
Treatment	N	R			R		
	S	0.13	0.11-0.16	<0.001	0.36	0.15-0.9	0.028
	C	0.46	0.38-0.55	<0.001	0.27	0.07-0.98	0.047
	R	0.61	0.42-0.87	0.006	2.3	0.59-9.01	0.233
	S+C	0.14	0.1-0.19	<0.001	0.21	0.03-1.51	0.121
	S+R	0.11	0.04-0.3	<0.001	NA	NA-NA	NA
	C+R	0.52	0.37-0.72	<0.001	NA	NA-NA	NA
	S+C+R	0.25	0.11-0.56	0.001	NA	NA-NA	NA
AFP	Negative	R			R		
	Positive	1.29	1.12-1.5	0.001	1.15	0.98-1.34	0.087
Bone	No	R			R		
metastasis	Yes	3.14	2.18-4.52	<0.001	1.07	0.7-1.65	0.751
Brain	No	R			R		
metastasis	Yes	35.59	4.9-258.48	<0.001	38.85	4.97 - 303.8	<0.001
Lung	No	R			R		
metastasis	Yes	4.33	3.23-5.81	<0.001	1.43	0.99 - 2.05	0.054

The C-index were 0.766, 0.765 and 0.801 in the training, internal validation and external validation set. The AUC values to predict 1-, 3- and 5-year OS was 0.843 (95% CI, 0.818-0.867), 0.849 (95% CI, 0.825-0.873) and 0.864 (95% CI, 0.840–0.888) in the training set, respectively (Figure 3D). In the internal validation set, the AUC values were 0.855 (95% CI, 0.820-0.891), 0.857 (95% CI, 0823-0.892) and 0.866 (95% CI, 0.831-0.901), respectively (Figure 3E). In the external validation set, the AUC values of the nomogram to predict 1-, 3- and 5-year OS were 0.869 (95% CI, 0.843-0.894), 0.864 (95% CI, 0.838-0.890) and 0.814 (95% CI, 0.678-0.950), respectively (Figure 3F).

### Old-age group

The 845 patients were randomly divided into the training set (n=587) and internal validation set (n=258). The demographic and clinicopathological characteristics were summarized in Supplementary Table 4. The 350 patients who diagnosed between 2015 and 2016 were selected as an external validation set. Based on the OS, the univariate Cox analysis in the training set showed that the significant indicators were sex, race, grade, T stage, N stage, M stage, AFP level, treatment and bone, brain, lung metastasis in Table 4. The model yielded the smallest AIC value (AIC=5612.22) when 9 variables, sex, race, grade, tumor size, T stage, N stage, M stage, AFP level, and treatment, were included into the multivariate Cox analysis. Then we constructed a reliable nomogram for the prediction of 1-, 3- and 5-year OS of PLCs in old-age group (Figure 2C).

**Table 4:** Univariate and multivariate Cox regression of the training set in old-age group.

**Characteristics**		**Univariate Cox analysis**			**Multivariate Cox analysis**		
		**HR**	**95%CI**	**p-value**	**HR**	**95%CI**	**p-value**
Sex	Male	R			R		
	Female	0.77	0.65-0.92	0.004	0.76	0.63-0.91	0.003
Race	White	R			R		
	Black	1.22	0.84-1.76	0.295	1.03	0.71-1.5	0.865
	Other	0.81	0.66-0.99	0.044	0.79	0.64-0.98	0.033
Marital	Married	R					
	Unmarried	1	0.84-1.2	0.96			
Histology	HCC	R					
	ICC	1.19	0.9-1.59	0.222			
Tumor.size	<5cm	R			R		
	5—10cm	1.4	1.14-1.71	0.001	1.27	0.99-1.61	0.055
	>10cm	1.89	1.51-2.38	<0.001	1.64	1.26-2.14	<0.001
Grade	Grade I	R			R		
	Grade II	0.87	0.71-1.07	0.192	1.05	0.85-1.3	0.655
	Grade III	1.44	1.14-1.81	0.002	1.9	1.48-2.44	<0.001
	Grade IV	1.25	0.69-2.27	0.456	2.33	1.22-4.45	0.011
T.stage	T1	R			R		
	T2	0.91	0.71-1.16	0.451	1.14	0.87-1.5	0.328
	T3	1.7	1.39-2.07	<0.001	1.31	1.06-1.63	0.014
	T4	1.52	1.06-2.18	0.022	1.14	0.76-1.69	0.527
N.stage	N0	R			R		
	N1	2.36	1.75-3.19	<0.001	1.32	0.94-1.86	0.106
M.stage	M0	R			R		
	M1	2.71	2.1-3.51	<0.001	1.79	1.2-2.66	0.004
Treatment	N	R			R		
	S	0.14	0.11-0.18	<0.001	0.22	0.05-0.94	0.042
	C	0.39	0.32-0.48	<0.001	0.32	0.26-0.4	<0.001
	R	0.41	0.28-0.6	<0.001	0.37	0.25-0.54	<0.001
	S+C	0.17	0.09-0.3	<0.001	0.21	0.04-1	0.050
	S+R	0.73	0.23-2.29	0.59	0.64	0.1-4.12	0.641
	C+R	0.38	0.23-0.64	<0.001	0.27	0.15-0.47	<0.001
	S+C+R	0.45	0.11-1.82	0.264	NA	NA-NA	NA
AFP	Negative	R			R		
	Positive	1.21	1.02-1.44	0.032	1.17	0.97-1.4	0.095
Bone metastasis	No	R			R		
	Yes	2.15	1.11-4.17	0.023	1.14	0.52-2.48	0.744
Brain metastasis	No	R			R		
	Yes	10.73	2.64-43.65	0.001	3.14	0.72-13.64	0.128
Lung metastasis	No	R			R		
	Yes	2.87	2.04-4.04	<0.001	1.07	0.66-1.73	0.779

The C-index were 0.781, 0.762 and 0.748 in the training, internal and external validation set. Respectively, the AUC values of the nomogram to predict 1-, 3- and 5-year OS were 0.856 (95% CI, 0.826-0.886), 0.879 (95% CI, 0.849-0.909) and 0.868 (95% CI, 0.830–0.905) in the training set (Figure 3G); 0.871 (95% CI, 0.829-0.914), 0.864 (95% CI, 0.817-0.910) and 0.878 (95% CI, 0.829-0.926) in the internal validation set (Figure 3H); and 0.800 (95% CI, 0.754-0.846), 0.817 (95% CI, 0.765-0.870) and 0.868 (95% CI, 0.776-0.960) in the external validation set (Figure 3I).

**Figure 4:**
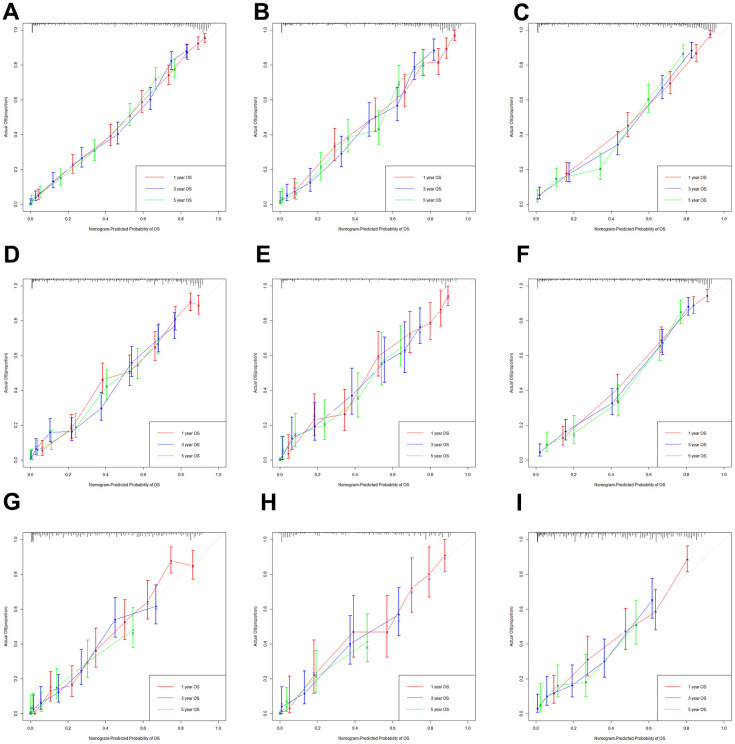
**The calibration curves based on nomogram.** The calibration curves based on nomogram in the training, internal and external validation set of low-age group (**A**–**C**), middle-age group (**D**–**F**) and old-age group (**G**–**I**).

**Figure 5:**
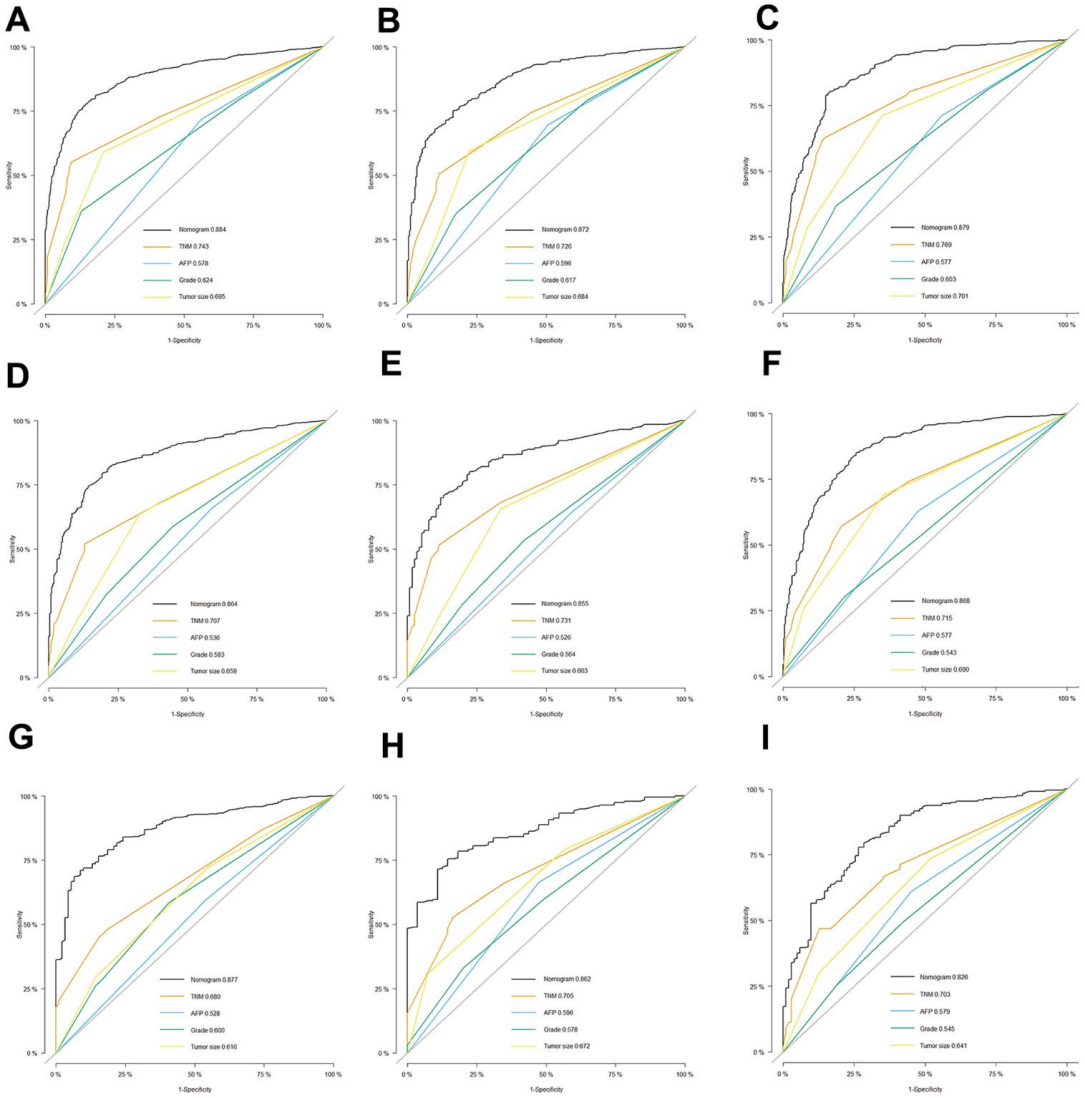
**Comparison of efficacy between our model and existing predicting factors.** The receiver operating characteristic (ROC) curves for the prediction of OS based on nomogram, TNM stage, AFP, grade and Tumor size in the training, internal and external validation set of low-age group (**A**–**C**), middle-age group (**D**–**F**) and old-age group (**G**–**I**).

**Figure 6:**
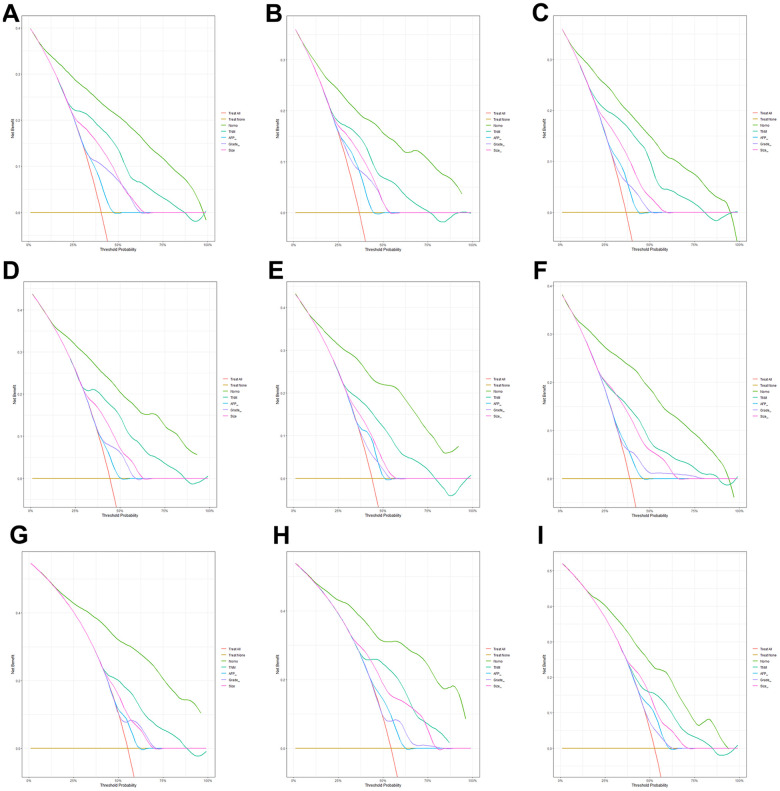
**Comparison of clinical utility between our model and existing predicting factors.** The decision curve analysis (DCA) curves for the prediction of OS based on nomogram, TNM stage, AFP, grade and Tumor size in the training, internal and external validation set of low-age group (**A**–**C**), middle-age group (**D**–**F**) and old-age group (**G**–**I**).

Furthermore, the calibration curves indicated an optimal agreement between the actual and predicted probability of OS in the training set (Figure 4A, 4D, 4G), internal set (Figure 4B, 4E, 4H) and external validation set (Figure 4C, 4F, 4I) in three age groups. This suggested our model was reliable and valid. We also observed that the AUC values of our nomogram model were superior than the independent factors such as TNM stage and grade, regardless of the age group (Figure 5). Additionally, the DCA curves demonstrated that the nomogram exhibited better clinical applicability and net benefits at different threshold probabilities compared to both the treat-all and treat-none schemes for guiding clinical intervention, irrespective of the age group. It also outperformed TNM stage and other independent factors. Meanwhile, patients in each age group can be categorized into three risk subgroups based on the total score (Figure 6). The Kaplan-Meier survival curve for OS showed significant difference in three risk subgroups in each age group (Figure 2D–2F). We also developed an accessible online dynamic page to simplify and visualize our model (http://124.222.247.135/).

### The effect of treatment in three age groups

**Figure 7:**
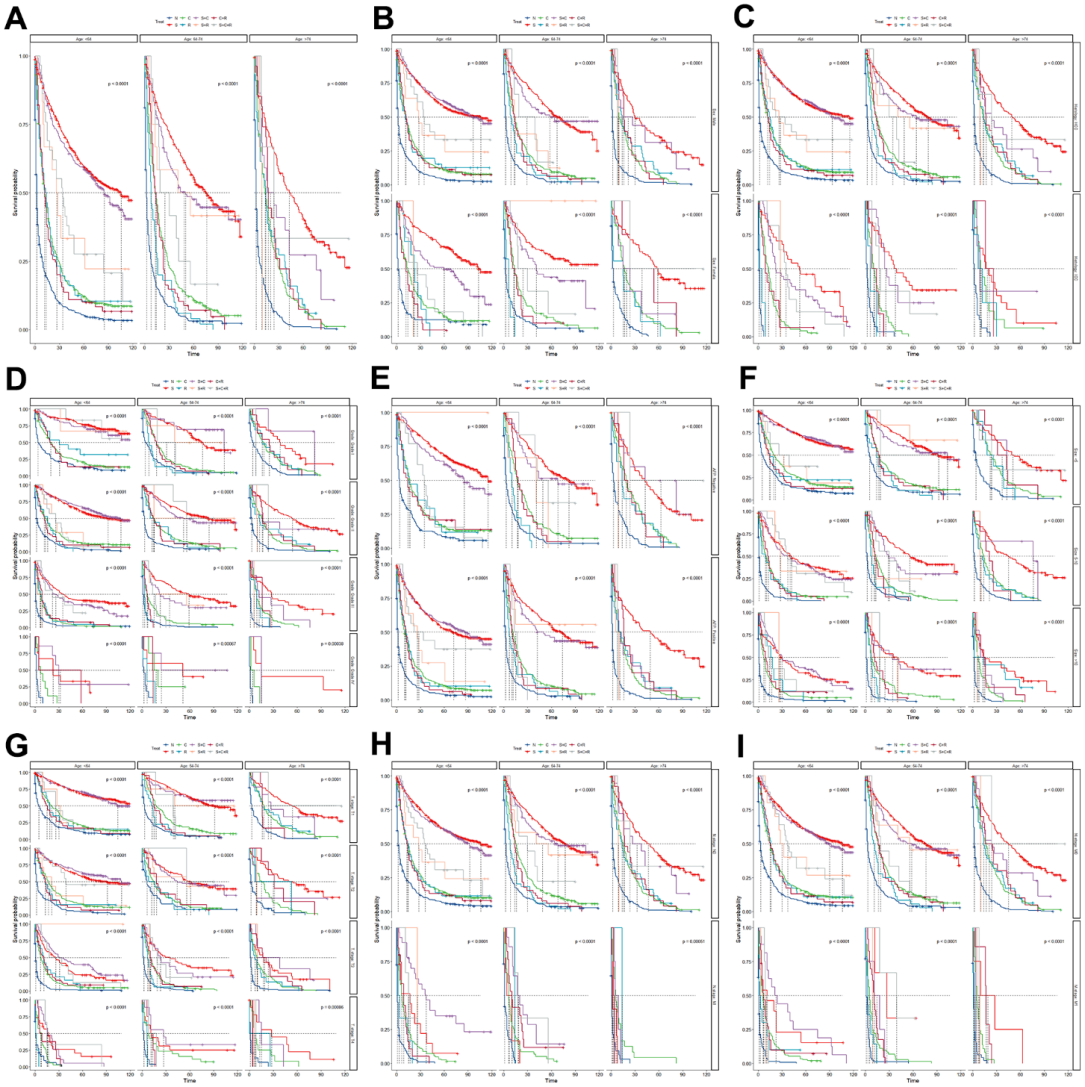
The Kaplan-Meier curve for overall survival (OS) among different treatments in three age groups (**A**) and further subgroup analysis in the sex (**B**), site (**C**), grade (**D**), AFP (**E**), tumor size (**F**), T stage (**G**), N stage (**H**) and M stage (**I**). N, no treatment; S, surgery alone; C, chemotherapy alone; R, radiation alone; S+C, surgery combined with chemotherapy; S+R, surgery combined with radiation; C+R, chemotherapy combined with radiation; and S+C+R, surgery combined with chemotherapy and radiation.

Overall, in the low-age group, the patients who received surgery alone (S) or surgery combined with chemotherapy (S+C) had the best prognosis, followed by surgery combined with radiation (S+R) and surgery combined with chemotherapy and radiation (S+C+R) (Figure 7A). Further subgroup analysis revealed that in the female (Figure 7B), ICC (Figure 7C), AFP negative subgroup (Figure 7E), the prognosis of patients who treated with surgery alone (S) was better than that of patients treated with surgery combined with chemotherapy (S+C). While, in the male, HCC, AFP positive subgroup, the prognosis of patients treated with surgery combined with chemotherapy (S+C) was better.

In the middle-age group, the prognosis of patients who received S was the best, followed by S+C and S+R (Figure 7A). Further subgroup analysis suggested in the female, ICC, poorly differentiated (Grade III/IV), T3, tumor size 5-10cm subgroup, the prognosis of patients who treated with S was better. Meanwhile, in the male, HCC, well differentiated (Grade I/II), T1/2, N0, M0 subgroup, the prognosis who treated with surgery alone and surgery combined with chemotherapy or radiation were similarly good (Figure 7).

The patients who treated with S had a better prognosis than other treatments in high-age group. After subgroup analysis, in the male, well differentiated (Grade I/II), tumor size <5cm subgroup, the patients who treated with S+C also had good prognosis. Meanwhile, the patients in ICC, N1, M1 subgroup had the poor prognosis regardless of the type of treatment received (Figure 7).

## DISCUSSION

Primary liver cancer (PLC) is a common malignant cancer and the third leading cause of cancer death in the world. Primary liver cancer is highly aggressive and prone to metastasis, resulting in poor prognosis for patients [2]. With the aggravation of the aging phenomenon, the age of diagnosis of primary liver cancer patients (PLCs) has been also increasing. Simultaneously, we have observed significant differences in prognosis among PLCs diagnosed at different age. Therefore, it is crucial to accurately predict the prognosis of patients with different diagnostic ages and to choose the appropriate treatment options.

In our study, we included age as a categorical variable and divided the study cohort into three groups based on the X-tile software: <64 years old, 64-74 years old and >74 years old. It was clear that we observed a significant difference in the 5-year OS between the three groups (31.0% vs 24.2% vs 13.0%, *P* < 0.001). We also found that although the proportion of female patients gradually increased with the growth of the age at diagnosis, in all three groups are most patients were male. In fact, in most countries, both the incidence and mortality rates of male patients with PLCs are 2 to 3 times higher than that of female. PLC ranks fifth in terms of global incidence and second in terms of mortality for men [2, 3]. This may be related to the sex hormone level. A study indicated that androgens/androgen receptor may promote the development of HBV-related HCC, potentially explaining the higher incidences of HCC in male than female. Conversely, estrogen/estrogen receptor may likely inhibit the formation and progression of HCC [21, 22]. Meanwhile, patients in old year group exhibited more cases of ICC, larger tumor size, worse T stage and a higher AFP positive rate, but no significant difference were observed in N, M stage, Grade. These may imply that different independent prognosis factors and their importance may differ in PLCs of different age groups. We included independent prognosis factors for each group based on the multivariate regression and AIC principle. The treatment, lung metastasis and bone metastasis were also included in the model as important prognosis factors to construct the nomogram. Brain metastasis was not included in the model because of fewer cases and significant bias [19, 23, 24].

Ultimately, we visualize our model as a Nomogram. With the ability to generate an individual numerical probability of a clinical event by integrating diverse prognostic and determinant variables, nomogram fulfill our drive towards personalized medicine. Meanwhile, rapid calculation through user-friendly digital interface improves accuracy and facilitates a better understanding of prognosis to aid in clinical decision-making [9]. The graphical nomogram is shown in Figure 2A, 2C, 2E, where each variable is listed separately and the corresponding number of points is assigned to a given variable magnitude. Then, the cumulative scores of all variables were compared with the outcome scale to obtain the outcome probability [8]. At the same time, web-based dynamic nomograms enable doctors to quickly and accurately predict prognosis, and even empower patients to do so themselves. In this study, we integrated the dynamic nomograms for three age groups into an open web page (http://124.222.247.135/). Users only need to enter the age at the diagnosis of liver cancer, and the page will automatically navigate to the corresponding age group model. Then, users can select the variables based on their actual situation and choose the desired time point for prediction, thereby obtaining the corresponding survival probability. For doctors, the nomogram enables them to make more accurate predictions for every liver cancer patient, especially patients of different ages. This aids in making more personalized decisions to enhance clinical benefits. For patients, the straightforward and user-friendly nomogram helps them gain a deeper understanding of their disease situation. This facilitates efficient doctor-patient communication and can also improve the traditional ‘active-passive’ doctor-patient relationship. Meanwhile, we evaluated the model with internal and external validation. The calibration curve displayed an optimal agreement between the predicted and actual survival, and the AUC curve suggested a higher discrimination compared to the traditional TNM stage and other indicators. The DCA curve suggested the nomogram exhibited good clinical applicability.

In addition, the choice of treatment was also a focal point of our attention. By survival analysis, we found that there was a better prognosis for S or S+C in the low age group, with the better prognosis for S in the middle age group, followed by S+C or S+R, and the best prognosis of S in the old age group. Overall, patients with PLC who treated with S+C had better prognosis and lower sensitivity to radiotherapy, consistent with existing literature [1, 25]. However, patients in the old-age group had the best prognosis for surgery alone, but a poor prognosis for combined treatments. This observation might be attributed to the weakened physical condition of elderly patients, making them less tolerant to the side effects of chemotherapy and radiotherapy, coupled with a relatively higher proportion of ICC in this age group. Meanwhile, further subgroup analysis suggested that the prognosis of PLCs in ICC subgroup in all age groups was worse than that of HCC due to the poor sensitivity to chemotherapy and radiotherapy, so surgery alone may benefit more, which consistent with previous studies [26]. The prognosis of S+C in male subgroup was significantly better than that of female, and the prognosis of female patients treated with S was better, which may be associated with the sex hormone level of female patients and make them insensitive to chemotherapy [21, 22]. The prognosis of S+C was better than S in AFP positive subgroup in the low age group. The elevated levels of AFP result from various somatic mutations resulting in the lack of an AFP inhibitor, which in turn may lead to faster and more aggressive growth of tumors [27]. There is a further link between vascular invasion in and AFP level in HCC according Franca AV et al. [28]. These may explain the better effect of the S+C for AFP positive patients. The prognosis was poor for patients with lung or bone metastases no matter what kind of treatment.

The highlight of this study is that we divided the primary liver cancer patients into three age groups according to the diagnosis age, explored the independent prognostic factors of patients in different age groups, and constructed a multivariate regression model, a visualize nomogram, to predict the prognosis of patients. And we developed an online web page integrating three models from our study. This provides a great help for the individualized treatment and clinical decision making of primary liver cancer patients. Secondly, we explored the impact of the treatment options in different age groups, and the results suggest that surgery alone may be the best treatment option for elderly liver cancer patients. Further results of subgroup analysis are good instructive for treatment selection of liver cancer patients.

However, our study also existed several limitations. Firstly, this was a retrospective study, it is subject to the inherent biases associated with this type of study design. Furthermore, limited by the SEER database, some indicators that may affect the prognosis of patients, such as whether HBV/HCV infection, whether drinking were not included in the study, the information about surgical treatment is not complete, such as surgical methods, cutting margin, portal vein invasion, which may make our model is not comprehensive. Unfortunately, the treatment information detailed in the database only includes surgery, chemotherapy and radiotherapy, and the information on immunotherapy and targeted therapy for patients is lacking in the database, which makes us inevitably missing this part of the information when making patient survival prediction and exploring the optimal treatment option. At the same time, the information in the SEER database is only derived from the United States, and it is not representative of the demographic and clinicopathological characteristics around the world. In the next study, we need to take a multicenter analysis to validate our model, while conducting prospective studies to include detailed influencing factors and treatment modalities to avoid these limitations and further optimize our results.

## CONCLUSIONS

The independent prognosis factors for PLC patients in different age group were identified in this study. We created an online page (http://124.222.247.135/) including three good nomograms that could be used to easily predict the survival probability of patients in different age groups and guide the making of clinical decision. The results of treatment analysis can provide reference about treatment choice for individual patients and revealed that the optimal therapeutic option for older patients with PLC was surgery alone.

## Supplementary Material

Supplementary Tables
